# Texas Health College Diplomas

**Published:** 1896-09

**Authors:** 

**Affiliations:** Beaumont, Texas


### The Texas Health College Diplomas Pass Current.

BEAUMONT, TEXAS, August 8, 1896.

*Editors Texas Medical Journal:*

Apropos of your reference to "Texas Health College" in your August issue of the JOURNAL, I must confess amusement at the assurance you give of speedy relief to physicians who are handicapped by graduates of this "Myth of the Mound," as you have happily advertised it. You cannot but be aware, my dear doctor, of the outcome of our fight with these charlatans, and I fear your promises of relief will fall flat when it comes to an issue. What if the Attorney-General does instruct district attorneys to prosecute these people? Don't you know that a grand jury will refuse to find a bill unless confronted by a witness from Mound who will testify that there is not, nor has there ever been a semblance of a medical school at Mound! Further, when the district attorney turns his attention to securing such a witness, he will find no funds appropriated for such purposes, and this, with your several days' investigation, will end the case, with the "Mound" doctor *mounded* higher than you are in the eyes of a great many. Hence, I am "agin" further agitation until we are certain of victory. The Southeast Texas Medical Society, at its last meeting, discussed this matter, and finally decided to raise a fund by assessing each member, for the purpose of defraying the expenses of a witness from the famous city, that we might overcome this defect of the law. Out of fairness to your readers who might become interested in the prosecution of these fellows, I think it but right that you give our sore experience at this end by telling the profession *what they continue to do it on.*

Yours, PRICE.

[Suggested: Write to Dr. J. T. Smith, Mound, Texas, for affidavits of old citizens that there is not, and never has been, such institution at Mound as the Texas Health College. It will cost nothing.—ED.]

